# Chronotopic encoding of emotional dimensions in the human brain assessed by FMRI

**DOI:** 10.1192/j.eurpsy.2021.361

**Published:** 2021-08-13

**Authors:** G. Lettieri, G. Handjaras, E. Ricciardi, P. Pietrini, L. Cecchetti

**Affiliations:** 1 Momilab - Sane Group, IMT School for Advanced Studies Lucca, Lucca, Italy; 2 Momilab, IMT School for Advanced Studies, Lucca, Italy

**Keywords:** affective timecourse, naturalistic stimulation, emotions, fMRI

## Abstract

**Introduction:**

Affective experiences vary as function of context, motivations and the unfolding of events. This temporal fundamental aspect of emotional processes is often disrupted in psychiatric conditions.

**Objectives:**

To investigate how the brain represents the association between affect and time, we combined fMRI and behavioral ratings during movie watching.

**Methods:**

Participants watched ‘Forrest Gump’ in the fMRI scanner (n=14, 6F). Data were preprocessed (see 10.1101/2020.06.06.137851v1) and average brain activity from 1000 regions was extracted. Independent subjects (n=12, 5F) provided continuous ratings of the intensity of their affective state while watching the same movie. Using PCA, we derived the first 3 affective dimensions (polarity, complexity, intensity; 10.1038/s41467-019-13599-z) and computed their time-varying correlation in windows from 5-1000tps. We identified the window size with the maximum between-subjects accordance and computed the inter-subject functional connectivity (10.1038/ncomms12141). For each region, we obtained connectivity strength and its association in time with changes in affective dimensions (p_Bonf_<0.05).

**Results:**

Fluctuations in connectivity strength of the right rMFG, precuneus, pSTS/TPJ, dmPFC, aINS and left pMTG were associated to polarity. Also, connectivity of the right IPS/SPL, SFG, dpreCS, IFGpOrb, OFC, precuneus, vpreCS and pSTS/TPJ followed the timecourse of perceived intensity of affect.

**Conclusions:**

Connectivity strength of default mode represents the pleasantness of the experience, whereas attention and control networks encode its intensity. Emotional descriptions converge in right temporoparietal and fronto-polar cortex, where the stream of affect is encoded in a chronotopic manner. These results expand our understanding of the neural correlates of emotional processing, a function severely affected by mental disorders.

**Disclosure:**

No significant relationships.

